# Linguistic markers of processing the first months of the pandemic COVID-19: a psycholinguistic analysis of Italian university students' diaries

**DOI:** 10.1007/s12144-023-04737-4

**Published:** 2023-05-19

**Authors:** G. Gandino, C. Civilotti, S. Finzi, M. Gaboardi, A. Guazzini, C. Novara, F. Procentese, M. Santinello, T. Sola, F. Veglia, E. M. Venera, G. Di Fini

**Affiliations:** 1 Department of Psychology, University of Turin, Turin, Italy; 2 Department of Developmental Psychology and Socialisation, University of Padua, Padua, Italy; 3 Department of Education, Languages, Interculture, Literatures and Psychology, Centre for the Study of Complex Systems (CSDC), University of Florence, Florence, Italy; 4 Department of Psychology, Educational Sciences and Human Movement, University of Palermo, Palermo, Italy; 5 University of Naples Federico II, Naples, Italy; 6 University of Chieti and Pescara, Chieti, Italy

**Keywords:** COVID-19, Photo diaries, LIWC, Linguistic markers

## Abstract

A longitudinal psycholinguistic study was conducted with 107 students from different Italian universities that produced daily photo-diary entries for two weeks, one at the beginning and the other at the end of the first Italian lockdown period, imposed in view of the rapid dissemination of COVID -19. The task was to take a daily photo accompanied by a short description (text). The texts accompanying the photos were analysed using Linguistic Inquiry and Word Count (LIWC) software to analyze linguistic markers representing psychological processes related to the experience of the pandemic and the lockdown, identifying potential changes in psycholinguistic variables useful for understanding the psychological impact of such harsh and extended restricted living conditions on Italian students. LIWC categories related to negation, anger, cognitive mechanisms, tentative discourse, past, and future increased statistically significantly between the two time points, while word count, prepositions, communication, leisure, and home decreased statistically significantly. While male participants used more articles at both time points, females used more words related to anxiety, social processes, past, and present at T1 and more related to insight at T2. Participants who lived with their partner showed higher scores on negative emotions, affect, positive feelings, anger, optimism, and certainty. Participants from southern Italy tended to describe their experiences from a collective and social perspective rather than an individual perspective. By identifying, discussing, and comparing these phenomena with the broader literature, a spotlight is shed for the first time on the psycholinguistic analysis of students at the national level who faced the first COVID -19 lockdown in Italy.

## Introduction

In Italy, the first phase of containment measures to combat the rapid spread of COVID-19 began on March 9, 2020 (D.P.C.M. 9 marzo 2020c); the measures were further tightened on March 22 (D.P.C.M. 22 marzo 2020a). This phase was also referred to as the lockdown phase or first lockdown, as the strictest isolation measures were applied during the following two years. For the most part, the population was confined to their homes, couldn't travel to another community, and were allowed to leave the house only for legitimate and documented reasons related to work, necessities (e.g., grocery shopping), or health. These were strict, sudden, and all-encompassing measures that severely affected most people's daily lives, especially their social relationships and activities. For almost two months, the extensive lockdown was maintained until May 4, when the government paved the way for a second phase in which containment measures were partially relaxed due to a steady decline in new cases of infection and hospitalizations (D.P.C.M. 26 aprile 2020b).

The literature indicates that these and similar containment measures have had a significant impact on the psychosocial well-being of many people around the world. To name a few aspects, significant increases in addiction, depression, anxiety, insomnia, and negative emotions in general have been reported (Dubey and Tripathi, 2020; Lakhan et al., 2020; Pieh et al., 2021; Prati, 2021). Although the physical isolation required to cope with the pandemic led to an increase in digital communication to mitigate the social and psychological consequences of the pandemic, and this mode of communication was adopted primarily by younger generations (Gómez-Galán et al., 2020), the literature showed that younger people reported more severe consequences and poorer well-being (Birditt et al., 2021; Gambin et al., 2021; Gualano et al., 2020; Pieh et al., 2021). Italy seems to be the country that suffers from the highest levels of Depression (Passavanti et al., 2021). In addition, an Italian survey showed that the greatest prevalence of high psychological impact was reported in the < 34 years' age group and in north Italy (Ferrucci et al., 2020). In this country the psychological impact influenced all the daily life aspects, requiring the implementation of different coping strategies to face the challenges of the lockdown (Gaboardi et al., 2022).

Even before the pandemic, university students were considered a category of mostly young people prone to increased psychological distress compared to the general population. Pre-pandemic literature reported higher levels of stress, anxiety, depression, sleep disturbances, and emotional instability (Liu et al., 2019; National Union of Students, 2015). Studies have shown that a significant proportion of university students experienced elevated levels of stress, suggesting that conditions of epidemic and confinement associated with the transition to distance learning may contribute to the development of posttraumatic stress symptoms in this population (Essadek & Rabeyron, 2020; Idoiaga et al., 2022; Li et al., 2021; Stamatis et al., 2021; von Keyserlingk et al., 2022). In addition, university students may have experienced increased levels of anxiety and depression (Chen and Lucock, 2022; Kaparounaki et al., 2020; Wang et al., 2020), which may lead to psychopathological conditions such as generalised anxiety disorder and major depression, with incidence in this population significantly higher than in previous years (Chirikov et al., 2020). In addition, the COVID -19 pandemic has increased addiction to social media, online games, and food among university students: the impact on personal, social, and psychological levels, as well as the link with drug use, cannot be neglected (Gómez-Galán et al., 2020). In Italy Quintiliani et al. (2022) reported that students’ stress significantly decreased learning and negatively affected psychological well-being.

Several studies have examined the impact of the pandemic and the lockdown by analysing people's written accounts, such as through social channels. Social media allows people to present themselves and their experiences to the world through the narration of opinions and stories (McAdams, 2018; Marzana et al., 2021). When unexpected, sudden and overwhelming events occur, storytelling is a tool to bring the extraordinary back to ordinary everyday life and share it with others (Bruner, 2004; McAdams & McLean, 2013). Some authors analyzed texts from social networks such as Twitter (Abdo et al., 2020; Essam and Abdo, 2021; Storey and O'Leary, 2022; Yu et al., 2021; Zhang et al., 2021). Others examined journal articles, personal stories, and search histories in search engines (Herat, 2020; Zhang et al., 2020). Finally, some studies used text compositions or a series of closed-ended questions (Kostruba, 2021). Literature consistently shows a predominance of themes closely associated with coronavirus and negative emotions. Tweeters have been found to exhibit elevated levels of fear, anger, and doubt despite high levels of analytical thinking (Abdo et al., 2020). Nonetheless, tweets also contain culturally specific language. For example, a study of Arabic tweets found that discussions about the pandemic were dominated primarily by psychological categories related to religion and health (Essam and Abdo, 2021). In addition, an analysis of news reports shows that the UK pays more attention to economics, while Sri Lankan newspapers prioritize educating the public about the severity of COVID -19 (Herat, 2020). Studies have noted a shift in language use during the pandemic, with an initial emphasis on information seeking and a subsequent increase in emotion, including anger, over time (Storey & O'Leary, 2022). In addition, emotions were found to vary throughout the day, with anxiety and anger more prevalent in the morning and afternoon, and depression more prevalent at night, although all emotions were generally more prominent in the afternoon and evening (Yu et al., 2021). The data collected suggest that forced self-isolation can lead to a breakdown in mental health (Herat, 2020) and that worsening depression and anxiety are strongly correlated with changes in Google search and YouTube behaviors (Zhang et al., 2020).

Pennebaker (2003) has shown how narrating traumatic and unpredictable events can help people sort through, process, and make sense of the experience. The analysis of narratives and language allows to explore psychological processes that occur during and across critical events (Tausczik & Pennebaker, 2010). As seen in clinical conversations, discourse styles that exhibit features such as rigidity, confusion, or lack of integration can serve as essential clues for a successful investigation of the patient's psychological processes (Veglia & Di Fini, 2017). On the other hand, the content of individual narratives usually converges into "narrative genres" or meaning clusters that support or sometimes impose thematic constraints or leitmotifs (Di Fini & Veglia, 2019). These concepts can be informative for researchers when considering the psychological functioning of people across the continuum between psychiatric patients and normative people.

The study of language and language markers in times of pandemics and restrictive policies continues to be the focus of many researchers. Psycholinguistic markers, including temporal, depersonalization, and affective process markers, were identified in the narratives to distinguish pre- and post-pandemic experiences. This confirms that the pandemic was a traumatic event (Kostruba, 2021). However, different age groups appeared to face unique challenges during the pandemic. Younger individuals found it more difficult to find appropriate spaces for self-isolation and to manage their overall well-being, while middle-aged individuals expressed greater concerns about balancing work and childcare. On the other hand, older individuals were more confident in their ability to take the necessary precautions to protect themselves.

Looking at Italy, only two psycholinguistic studies seem to be available for this time window, focusing on the Italian territory, and none of them were longitudinal, focused on students, or analyzed material other than tweets or similar social media posts: one looked at the Italian region of Lombardy and the Chinese region of Wuhan (Su et al., 2020), two of the first regions to be sealed off globally; the other examined a large number of Italian COVID-19 tweets written by the general population and used LIWC2015 to investigate expressed emotions, thinking and somatosensory processes (Monzani et al., 2021). Overall, these studies demonstrate the usefulness of language analysis for understanding public attitudes and emotions during a pandemic, particularly through online data, which has proven to be a rich source of data for researchers interested in studying public perceptions and emotions during times of crisis.

However, in examining the literature, we have found that there is a gap in psycholinguistic research that does not refer to big data collected on the Internet, but to the private, internal experiences that people (in our case Italians) have day by day under strict constraints, and to their evolution over time. This represents a significant gap in psycholinguistic research and highlights the need for a more detailed analysis of individual experiences. To fill this gap, we focused on a different research material: multiple narratives produced daily as a description of a meaningful photograph for the participant by Italian university students who faced the most restrictive measures imposed by the Italian government to combat the spread of COVID -19 during the initial response period described above. Knowing the strengths of the Linguistic Inquiry Word Count (LIWC) analysis in terms of extensibility and validity, we chose to analyze these texts. LIWC analysis allows us to identify and quantify the use of different word categories in the text, such as emotional, social, and cognitive language. This can provide valuable insight into the psychological and emotional state of individuals as well as the cultural and social context in which they live (Tausczik & Pennebaker, 2010; Pennebaker and Stone, 2003). As an experimental task, we chose the photo diary method. Texts were created as descriptions of a photograph taken each day for a week; the entire weekly procedure was repeated a second week to have two time points for comparison, one just before the start of the lockdown (T1 = 25 to March 31, 2020) and the other just before its end (T2 = 22 to April 28, 2020). We hypothesized that the pandemic and the isolation influenced the students in the choice of language to express their daily experience. We expected that the progressive easing of the rigid restrictive measures would be reflected in a change in the use of language. Since the situation regarding the pandemic in Italy wasn't uniform and the northern regions were more affected by the virus than others (Ciminelli & Garcia-Mandicó, 2020; Rondanelli et al., 2021; Statista Research Department, 2021), we wanted to investigate the differences between the parts of Italy in terms of psycholinguistic categories. The project involved two universities from each part of the country: North, Central and South, but often people lived in a different city at the time of lockdown; so we considered the place of residence during the lockdown. Accordingly, we hypothesized the existence of differences between the Italian areas also in the use of language. Considering the influences of possible roommates/family members during isolation, we also focused on differences in language use according to residential status at the time of the lockdown. Given the obligation of closer interactions during the lockdown, we expected those who lived with someone to report more emotional descriptions (positive or negative) of those who were alone.

## Methods

### Participants

Participating students were recruited on a voluntary basis, emphasising that there were no bonuses or compensation, financial or curricular. The sample was recruited by presenting the research during the Community Psychology and Clinical Psychology courses in each University. The study could start after the completion of the informed consent form by the participants and the prior approval by the Ethics Committee of the University2. Participants took part in the study voluntarily without financial compensation.

Our convenience sample consisted of 107 university students (93 women and 14 men) with a mean age of 23.72 years (sd = 5.17) attending six different Italian universities – University1, University2, University3, University4, University5 and University6. The different participation of women and men in the study reflects the proportions in Italian universities, as well as the age of the participants, which tends to be younger and corresponds to the age distribution reported by the Italian public research organisation Istat (2016).

The characteristics of our sample in terms of university, geographic location, and residential status are presented in Table 1.

**Table 1:** Characteristics of the students involved in the research (*N* = 107)

Characteristics of the participants	*n* (%)
University of belonging	
- University4	5 (4.67)
- University3	22 (20.56)
- University5	36 (33.64)
- University2	10 (9.34)
- University6	14 (13.08)
- University1	20 (18.69)
Geographical localization during lockdown	
- North	29 (27.10)
- Center	23 (21.49)
- South	55 (51.40)
Housing status	
- Alone	4 (3.74)
- With partner	6 (5.61)
- With family	71 (66.35)
- With friends/roommates	4 (3.74)
- Did not provide this information	22 (20.56)

### Procedure

 In the present study we chose to use the photo diary method. Each participant was asked to take a photograph every day for a week that reflected his or her mood during the day he or she was confined to home. Each photo was to be captioned and briefly described (no more than 400 words) in response to the following questions: "Describe the content of the photo" (a); "Why did you take this photo?" (b); "What did you want to represent with this photo?" (c); "How does it relate to your experience during this time of health distress?" (d). The photo with the text was due by midnight on the day it was taken. Participants sent via e-mail every day all their materials (photo, title, and brief description) to the unit contact professor in a Word file. Frequent contacts between professors and course participants favoured continuity of participation in the study.

The activity just described was performed daily by participants for two weeks, between March 25 and 31, 2020 (T1, third week of the lockdown in Italy) and between April 22 and 28, 2020 (T2, penultimate week of the lockdown).

Socio-demographic data such as gender, age and housing status were collected through an ad hoc form. Furthermore, participants were asked to indicate the place where they lived during the lockdown (geographical localization), then recoded in terms of Italian geographic areas, Northwest, Northeast, Center and South.

### Data analytic strategy

To perform a reliable psycholinguistic analysis, we chose the Linguistic Inquiry and Word Count (LIWC) software, whose latest version (LIWC-15; Pennebaker et al., 2015) can work with text input in Italian (see also: Alparone et al., 2004). The software analyses the text by computing additive values and proportions in two ways: it tracks several summary variables such as "Word Count," "Cognitive processes," "Emotional Tone," that synthesise information at a macro level; it uses over a hundred dictionaries to categorise the text word by word, calculating the proportions of terms that fall into each dictionary. These dictionaries include function words – such as pronouns, prepositions, or adverbs – and content words related to psychological processes – e.g., "sensations," "cognition," "affect," "social processes" – and to dominant life themes and thought topics and tendencies – e.g., "culture," "space," "perception," "time orientation" (Pennebaker et al., 2015).

LIWC has been used, validated, and tested for accuracy countless times in its successive versions spanning more than twenty years, leading the literature to agree on a very positive evaluation of the software (e.g., Pennebaker et al., 2015; Zhao et al., 2016). After collecting all the photo presentations made by the participants during the two phases (for each student 7 texts for T1 and 7 texts for T2), titles and descriptions were combined into a single unit that included all the texts produced by each participant. We chose to aggregate the responses for three questions into one single text because these were already naturally united by the participants in a single description that accompanied each photo. In fact, the students did not provide a text divided into points, but gathered the answers into a more fluid and discursive composition. At the end of this process, each participant was associated with two texts, one for each time point.

Four statistical models were conducted. A paired-samples t-test was used to determine if there was a statistically significant mean difference between T1 and T2 with respect to LIWC categories. Because normality assumptions were not met, the Mann–Whitney U test was used to identify and explore significant differences in LIWC categories between men and women at both T1 and T2. A one-way analysis of variance with Tukey post hoc tests was performed for the LIWC categories to examine differences between groups from different parts of Italy. A Kruskal–Wallis test was chosen over an ANOVA due to the smaller sample sizes in participants who lived alone or with other individuals. As for social science research a significance level of *p* < 0.05 is acceptable (Gall et al., 2007), we used a two-tailed alpha of *p* < 0.05 to test for significance. Statistical analyses were performed using SPSS version 27.0.

## Results

Students provided one photo each day for seven days at T1 and seven days at T2. On average, students used 196.6 (SD = 81.9) words each day at T1, and 181.2 (SD = 88.7) words each day at T2.

The paired-samples t-test showed which psycholinguistic categories had statistically significant differences between the two time points.

The categories *negations*, *anger*, *cognitive mechanisms*, *tentative*, *past*, *future* reported an increase between T_1_ and T_2_; the categories *word count*, *prepositions*, *communication*, *leisure* and *home* instead reported a significant decrease (Table 2).

**Table 2:** Means and standard deviations of LIWC categories at T1 and T2, with significant values (in bold) related to differences between the two times

	Mean (T1)	SD (T1)	Mean (T2)	SD (T2)	*t*	*p*
Word count Pronouns 1^st^ person singular 1^st^ person plural Self 2^nd^ person Others Negations Assent Articles Prepositions Affect	1376.5 7.6 3.2 0.7 1.6 0 0.2 1.7 0.1 10.1 11.4 4.2	573 1.5 1.3 0.4 0.5 0 0.2 0.6 0.1 1.2 1.3 1.1	1268.5 7.6 3.2 0.7 1.5 0 0.2 1.9 0.1 10.0 11.0 4.3	607 1.4 1.2 0.4 0.5 0 0.2 0.8 0.1 1.2 1.2 1.1	4.3 0.5 0.7 -0.03 1.6 -1.6 -0.9 -2.6 -0.8 1.5 3.7 -0.8	**0.00** 0.6 0.51 0.97 0.12 0.11 0.32 **0.01** 0.43 0.13 **0.01** 0.42
Positive sensations Positive emotion Negative emotion Anxiety Anger Sadness	2.5 0.7 1.6 0.3 0.3 0.8	0.8 0.4 0.6 0.4 0.2 0.4	2.6 0.8 1.5 0.3 0.4 0.7	0.9 0.5 0.7 0.3 0.2 0.4	-1.6 -0.9 0.7 -0.7 -2.0 1.4	0.1 0.33 0.46 0.5 **0.04** 0.16
Cognitive Processes Causation Insight Discrepancy Inhibition Tentative	5.6 1.9 1.8 0.2 0.2 2.3	1.2 0.7 0.5 0.2 0.2 0.7	5.8 1.9 1.9 0.2 0.2 2.5	1.2 0.7 0.6 0.2 0.2 0.8	-2.0 -0.9 -0.7 -1.2 -0.02 -2.6	**0.04** 0.33 0.5 0.23 0.98 **0.01**
Certainty Perception Visual	1.3 1.3 0.5	0.5 0.5 0.3	1.3 1.3 0.5	0.5 0.5 0.3	0.6 -0.0 0.5	0.56 0.99 0.62
Auditory Feeling Social processes Communication	0.3 0.4 3.2 0.8	0.2 0.3 0.9 0.3	0.3 0.4 3.1 0.7	0.2 0.3 1.0 0.4	0.1 -0.2 1.0 2.3	0.89 0.82 0.29 **0.04**
Friends Family Humans	0.2 0.4 0.5	0.2 0.3 0.3	0.2 0.4 0.5	0.2 0.4 0.3	0.3 0.2 -0.1	0.74 0.82 0.95
Time Past focus Present focus Future focus Space Up Down Inclusion Exclusion Motion Occupation	5.1 1.4 8.2 0.1 1.2 0.2 0.0 2.9 4.7 1.1 1.0	1.0 0.6 1.3 0.1 0.4 0.2 0.1 0.8 1.0 0.5 0.4	5.3 1.8 8.3 0.2 1.2 0.2 0.0 2.8 4.7 1.1 0.9	1.2 0.7 1.4 0.2 0.4 0.2 0.1 1.0 1.1 0.5 0.4	-1.9 -6.2 -0.5 -2.8 -0.8 -0.3 -1.2 1.4 -1.9 0.2 1.7	0.06 **0.00** 0.6 **0.00** 0.43 0.79 0.24 0.16 0.85 0.81 0.08
School Work Achievement Leisure Home Sport TV_it Music Money Metaphysics Religion Death Physical Body Sexual Eat	0.3 0.2 0.6 1.2 1.0 0.0 0.1 0.1 0.1 0.2 0.2 0.0 0.8 0.5 0.1 0.2	0.3 0.1 0.3 0.5 0.5 0.1 0.1 0.1 0.1 0.2 0.2 0.1 0.4 0.3 0.1 0.2	0.3 0.2 0.7 1.0 0.9 0.0 0.1 0.1 0.1 0.2 0.1 0.1 0.8 0.5 0.1 0.2	0.3 0.2 0.4 0.5 0.5 0.1 0.1 0.1 0.1 0.2 0.1 0.1 0.4 0.3 0.1 0.3	1.9 1.3 -0.7 2.8 3.1 -0.4 -0.1 -0.3 -0.1 1.1 1.8 -0.8 -0.3 -0.2 0.3 -0.1	0.05 0.19 0.5 **0.00** **0.00** 0.66 0.9 0.7 0.9 0.26 0.07 0.4 0.76 0.86 0.73 0.9
Sleep	0.1	0.1	0.1	0.1	-1.5	0.13
Health	0.0	0.1	0.0	0.1	0.2	0.86

The Mann–Whitney U test was used to evaluate the difference between men and women in the use of the different linguistic categories in the two time periods (Table 3). The results show that women generally used more words than men at both survey time points, while men used articles more frequently than women. At T1, women reported higher levels of *anxiety*-related words, *social processes*, *past* and *present* compared to men. At T2, however, women reported higher levels of *positive emotions* and *motion*-related words than men.

**Table 3:** Mann–Whitney test with significant values (bold) to compare males and females based on the LIWC categories at T1 and T2 (*N* = 107; *F* = 93, *M* = 14)

	Gender	Mean Rank (T1)	Sum of Rank (T1)	MWU	p	Mean Rank (T2)	Sum of Rank (T2)	MWU	*p*
Word count	F	56.22	5228	445	**0.05**	56.39	5244	429	**0.04**
	M	39.29	550			38.14	534		
Pronouns	F	56.13	5220	453	0.06	54.86	5102	571	0.46
	M	39.86	558			48.29	676		
1^st^ person singular	F	55.06	5121	552	0.36	54.84	5100.5	572.5	0.47
	M	46.93	657			48.39	677.5		
1^st^ person plural	F	55.81	5190	483	0.12	53.96	5018.5	647.5	0.97
	M	42.00	588			54.25	759.5		
Self	F	56.18	5224	448.5	0.06	55.06	5121	552	0.36
	M	39.54	553			46.93	657		
2^nd^ person	F	54.23	5043	630	0.49	54.68	5085	588	0.23
	M	52.50	735			49.5	693		
Others	F	53.43	4969	598	0.62	56.37	5242.5	430.5	**0.04**
	M	57.79	809			38.25	535.5		
Negations	F	55.85	5194	479	0.11	54.89	5104.5	568.5	0.45
	M	41.71	584			48.11	673.5		
Assent	F	54.27	5047	626	0.80	53.07	4935.5	564.5	0.39
	M	52.21	731			60.18	842.5		
Articles	F	51.47	4787	416	**0.03**	51.5	4789.5	418.5	**0.03**
	M	70.79	991			70.61	988.5		
Prepositions	F	53.53	4978	607	0.68	55.04	5119	554	0.37
	M	57.14	800			47.07	659		
Affect	F	55.03	5118	555	0.37	55.06	5120.5	552.5	0.36
	M	47.14	660			46.96	657.5		
Positive sensations	F	53.78	5001	630.5	0.85	56.01	5209	464	0.08
	M	55.46	660			40.64	569		
Optimism	F	51.89	4825.5	454.5	0.06	53.67	4991.5	620.5	0.78
	M	68.04	952.5			56.18	786.5		
Positive emotion	F	55.87	5196	477	0.10	56.44	5249	424	**0.04**
	M	41.57	582			37.79	529		
Negative emotion	F	55.88	5196.5	476.5	0.10	54	5022	651	1
	M	41.54	581.5			54	756		
Anxiety	F	57.40	5338.5	334.5	**0.00**	55.6	5171	502	0.17
	M	31.39	439.5			43.36	607		
Anger	F	54.34	5053.5	619.5	0.77	53.65	4989.5	618.5	0.76
	M	51.75	724.5			56.32	788.5		
Sadness	F	54.67	5084	589	0.56	54.15	5036	637	0.89
	M	49.57	694			53	742		
Cognitive Processes	F	54.08	5029.5	643.5	0.94	54.99	5114.5	558.5	0.39
	M	53.46	748.5			47.39	663.5		
Causation	F	53.69	4993	622	0.78	53.3	4957	586	0.55
	M	56.07	785			58.64	821		
Insight	F	53.22	4949.5	578.5	0.50	56.16	5223	450	0.06
	M	59.18	828.5			39.64	555		
Discrepancy	F	55.41	5153	520	0.22	54.18	5038.5	634.5	0.88
	M	44.64	625			52.82	739.5		
Inhibition	F	53.69	4993.5	622.5	0.79	53.41	4967.5	596.5	0.61
	M	56.04	784.5			57.89	810.5		
Tentative	F	55.97	5205	468	0.09	54.98	5113.5	559.5	0.39
	M	40.93	573			47.46	664.5		
Certainty	F	54.33	5052.5	620.5	0.77	53.79	5002.5	631.5	0.86
	M	51.82	725.5			55.39	775.5		
Perception	F	54.18	5039	634	0.87	54.97	5112.5	560.5	0.40
	M	52.79	739			47.54	665.5		
Visual	F	52.51	4883	512	0.19	53.4	4966.5	595.5	0.61
	M	63.93	895			57.96	811.5		
Auditory	F	54.45	5064	609	0.69	56.05	5212.5	460.5	0.08
	M	51.00	714			40.39	565.5		
Feeling	F	55.76	5186	487	0.13	54.94	5109.5	563.5	0.42
	M	42.29	592			47.75	668.5		
Social processes	F	56.72	5275	398	**0.01**	54.6	5078	595	0.60
	M	35.93	503			50	700		
Communication	F	56.04	5212	461	0.07	54.07	5028.5	644.5	0.95
	M	40.43	566			53.54	749.5		
Friends	F	55.22	5135	538	0.29	55.7	5180	493	0.14
	M	45.93	643			42.71	598		
Family	F	55.03	5117.5	555.5	0.37	55.04	5119	554	0.37
	M	47.18	660.5			47.07	659		
Humans	F	55.22	5135.5	537.5	0.29	53.46	4971.5	600.5	0.64
	M	45.89	642.5			57.61	806.5		
Time	F	53.35	4962	591	0.57	55.42	5154	519	0.22
	M	58.29	816			44.57	624		
Past focus	F	56.61	5265	408	**0.02**	55.31	5143.5	529.5	0.26
	M	36.64	513			45.32	634.5		
Present focus	F	56.59	5263.5	410.5	**0.02**	54.74	5091	582	0.52
	M	36.82	515.5			49.07	687		
Future focus	F	53.93	5015.5	644.5	0.95	52.78	4909	538	0.29
	M	54.46	762.5			62.07	869		
Space	F	54.93	5108.5	564.5	0.42	55.27	5140	533	0.27
	M	47.82	669.5			45.57	638		
Up	F	52.43	4876	505	0.17	53.32	4959	588	0.56
	M	64.43	902			58.5	819		
Down	F	52.74	4905	534	0.22	54.4	5059.5	613.5	0.70
	M	62.36	873			51.32	718.5		
Inclusion	F	54.90	5105.5	567.5	0.44	55.55	5166.5	506.5	0.18
	M	48.04	672.5			43.68	611.5		
Exclusion	F	55.28	5141.5	531.5	0.27	55.06	5121	552	0.36
	M	45.46	636.5			46.93	657		
Motion	F	52.23	4857	486	0.12	56.24	5230	443	**0.05**
	M	65.79	921			39.14	548		
Occupation	F	53.68	4992.5	621.5	0.78	54.44	5063	610	0.70
	M	56.11	785.5			51.07	715		
School	F	52.70	4901	530	0.26	54.85	5101.5	571.5	0.46
	M	62.64	877			48.32	676.5		
Work	F	53.35	4962	591	0.57	52.92	4922	551	0.35
	M	58.29	816			61.14	856		
Achievement	F	55.45	5157	516	0.21	55.33	5145.5	527.5	0.25
	M	44.36	621			45.18	632.5		
Leisure	F	53.02	4931	560	0.4	53.41	4967	596	0.61
	M	60.50	847			57.93	811		
Home	F	53.59	4983.5	612.5	0.72	53	4929	558	0.39
	M	56.75	794.5			60.64	849		
Sport	F	53.08	4936	565	0.34	54.26	5046	627	0.79
	M	60.14	842			52.29	732		
TV	F	53.03	4932	561	0.36	53.75	4998.5	627.5	0.80
	M	60.43	846			55.68	779.5		
Music	F	54.31	5051	622	0.76	54.12	5033	640	0.91
	M	51.93	727			53.21	745		
Money	F	52.63	4895	524	0.22	53.08	4936	565	0.41
	M	63.07	883			60.14	842		
Metaphysics	F	52.90	4919.5	548.5	0.34	53.99	5021.5	650.5	0.99
	M	61.32	858.5			54.04	756.5		
Religion	F	53.29	4956	585	0.53	53.45	4970.5	599.5	0.63
	M	58.71	822			57.68	807.5		
Death	F	53.81	5004.5	633.5	0.84	55.02	5117	556	0.31
	M	55.25	773.5			47.21	661		
Physical	F	54.61	5078.5	594.5	0.60	53.73	4996.5	625.5	0.81
	M	49.96	699.5			55.82	781.5		
Body	F	54.56	5074	599	0.63	55.08	5122.5	550.5	0.35
	M	50.29	704			46.82	655.5		
Sexual	F	54.61	5079	594	0.58	54.99	5114.5	558.5	0.38
	M	49.93	699			47.39	663.5		
Eat	F	54.85	5101.5	571.5	0.46	55.92	5201	472	0.09
	M	48.32	676.5			41.21	577		
Sleep	F	52.60	4891.5	520.5	0.21	52.03	4839	468	0.08
	M	63.32	886.5			67.07	939		
Health	F	53.40	4966.5	595.5	0.54	55.66	5176.5	496.5	0.07
	M	57.96	811.5			42.96	601.5		

Independent Sample Kruskal–Wallis test was conducted to analyze any differences in language use as a function of students' residential status at the time of the lockdown (Table 4). At T_1_, words included into the macro-category *affect* and to the categories *1*^*st*^* person plural*, *positive sensations*, *optimism*, *anger* showed statistically significant differences between the different living conditions. Specifically, a post-hoc Mann–Whitney test was performed and revealed significant differences between participants who lived with someone (partner, family, friends/roommates) at the time of the lockdown compared to those who lived alone in all categories. In most of these LIWC categories, participants who lived with a partner had higher levels than the other participants. At T_2_, *prepositions* were used more frequently by participants who lived with friends/roommates than by others.

**Table 4:** Independent Sample Kruskal–Wallis test on group differences (living alone, with partner, with family, with friends) in several word use categories, at T1 and T2 (*N* = 85)

LIWC category at T1	Alone	With partner	With family	With friends	*H*	Post hoc
	M(DS)	M(DS)	M(DS)	M(DS)		
1^st^ person plural	0.32 (0.07)	0.6 (0.39)	0.8 (0.44)	0.39 (0.34)	8.64^*^	A < Fa
Affect	3.4 (0.29)	6.33 (1.12)	4.24 (0.94)	3.64 (1.73)	17.08^***^	A < Fr < Fa < P
Positive sensations	1.76 (0.21)	3.59 (1.18)	2.5 (0.78)	2.31 (0.62)	12.28^**^	A < Fa < P
Anger	0.09 (0.12)	0.64 (0.48)	0.19 (0.17)	0.14 (0.11)	12.04^**^	A < Fr < Fa < P
Optimism	0.43 (0.21)	1.15 (0.26)	0.74 (0.36)	0.69 (0.47)	10.27^**^	A < Fa < P
LIWC category at T2	Alone	With partner	With family	With friends	*H*	Post hoc
	M(DS)	M(DS)	M(DS)	M(DS)		
Prepositions	12.21 (0.34)	10.13 (0.92)	10.81 (1.17)	12.49 (0.42)	13.79^**^	P < Fa < A < Fr

* *p* < 0.05; **p < 0.01; ***p < 0.001

A one-way ANOVA was performed to analyze any differences in language use depending on the place of residence in different parts of Italy (northwest, northeast, central, and south) (Table 5). At T1, the results show differences in the categories *1st person plural*, *certainty*, *social processes*. A post-hoc Tukey analysis showed that participants who lived in the northwest of Italy used fewer words from these categories than participants from the south of Italy. At T2, the categories *1st person plural*, *positive emotions*, *insight*, and *social processes* showed statistically significant differences. Specifically, participants who lived in the northwest of Italy used fewer *1st person plural* words, insight, and *social processes* than participants from the south or center of Italy. Participants who lived in the center of Italy used fewer words included in the *positive emotions* category than participants from the south or center of Italy.

**Table 5:** Significant group differences (residing in the northwest, northeast, central, south of Italy) in several word use categories, at T1 and T2 (*N* = 107)

LIWC category at T1	NW	NE	C	S	*F*	Post hoc
	M(DS)	M(DS)	M(DS)	M(DS)		
1^st^ person plural	0.46 (0.38)	0.97 (0.35)	0.66 (0.40)	0.85 (0.42)	5.98^***^	NW < S
Certainty	1.1 (0.43)	1.05 (0.20)	1.37 (0.43)	1.45 (0.48)	4.34^**^	NW < S
Social processes	2.76 (1.06)	3.49 (0.37)	3.11 (0.75)	3.48 (0.96)	3.68^***^	NW < S
LIWC category at T2	NW	NE	C	S	*F*	Post hoc
	M(DS)	M(DS)	M(DS)	M(DS)		
1^st^ person plural	0.43 (0.32)	0.8 (0.23)	0.68 (0.32)	0.88 (0.47)	7.03^***^	NW < S
Positive emotions	0.68 (0.48)	0.78 (0.23)	0.59 (0.41)	0.93 (0.45)	3.65^*^	C < S
Insight	2.32 (0.81)	1.73 (0.48)	1.68 (0.57)	1.93 (0.68)	3.67^*^	NW > C
Social processes	2.52 (1.24)	3.15 (1.1)	3.34 (0.85)	3.32 (0.89)	3.93^*^	NW < S < C

## Discussion

Paired samples t-test revealed 11 significant variations out of a total of 65 pairs formed by the psycholinguistic categories detected in both time points T_1_ and T_2_. Mean values with statistically significant differences were obtained for the following categories: *word count, negation, prepositions, anger, cognitive mechanisms, tentative, communication, past, future, leisure, home*. Of these categories, *negation, anger, cognitive mechanisms, tentative, past,* and *future* experienced an increase between T_1_ and T_2_; on the other hand, *word count, prepositions, communication, leisure,* and *home* experienced a decrease.

The results obtained are mostly consistent with what has been highlighted in the literature. In some studies, *anger* words (e.g., *hate, kill, annoy*) were found to be more frequent during the lockdown, with a significant increase compared to the pre-pandemic period (Chew et al., 2020; Yu et al., 2021). In our sample anger also increased between T1 and T2, confirming the hypothesis of literature that people show a change in language from an initial accent on information seeking to an increase in the expression of anger (Storey & O'Leary, 2022). There has also been an increase in the use of words that express *negation*, which were primarily associated with mood lowering in Pennebaker and Stone’s (2004) study.

It seems interesting to us that words expressing an increase in *negative emotions* (e.g., *hurt, ugly, nasty*) refer to two opposite attitudes. The first is more active and involves a relaxation of inhibitions and a change in response to elements perceived as threats or provocations (Cabral et al., 2016). The second is more passive and consists of a rejection of the painful or traumatic event, superior to the ability to process it (Freud, 1925). Two strategies for submitting to an imposed restriction on personal freedom for which there is no precedent to refer to. This consideration becomes even more interesting when we consider that it also extends to the category of *cognitive mechanisms* (e.g., *cause, know, ought*). Some studies argue that the use of words that fall into this category may express a higher level of cognitive processing and thought structuring (Su et al., 2020). We can therefore hypothesize that subjects use more cognitive processes over time to contain disturbing emotions (anger, dejection), which tend to increase. According to the literature, the use of cognitive words testifies to the presence of a process of meta-reflection on an unfavorable experience in order to make sense of it and distance oneself from the feeling of helplessness (Martino et al., 2015; Gandino et al., 2020). In addition, recounting traumatic events can be a way to make sense of traumatic experiences (Pennebaker, 2003) and help people reduce rigidity, confusion, and lack of integration associated with maladaptive forms of acceptance and adjustment to reality (Veglia and Fini, 2017; Gandino et al., 2022). This also seems consistent with the study of Procentese et al. (2021), who highlighted coping strategies adopted in diary narratives during the first months of the pandemic in terms of meaning-making as adaptation, redefinition of primary as well as broader social relationships.

It is also interesting to note the increase between T_1_ and T_2_ of the *past* and *future* categories, even though the task asked subjects to write diaries, which caused them to focus mainly on the present. The literature suggests that the use of verbs in the past tense indicates trauma processing and greater psychological distance from events, whereas verbs in the future tense are generally associated with a positive view of the situation experienced (Tausczik and Pennebaker, 2010; Kostruba, 2021). However, the increase in both categories forces us to reflect on the difficulty of remaining with the mind anchored in the present, which we try to escape from with increasing duration of forced confinement by taking refuge in memories of the past or future prospects. The use of the category *tentative* (e.g., *maybe, perhaps*) is consistent with this hypothesis, which could serve to express hope, presumably in the sense of a return to a normal situation: as the literature on storytelling has shown (Bruner, 2004; McAdams & McLean, 2013), narratives have the function of returning the unexpected, traumatic events to normal life. In fact, T2 coincides with the penultimate week of restrictions and is thus close to the resolution of the lockdown. The idea that the isolation will soon end may have shifted the discourse into the future rather than anchoring it in the present.

Regarding the decline in the *prepositions*, *communication*, *leisure*, and *home* categories, it is interesting to compare our results with those of the study by Su et al. (2020). Their work focused on comparing the impact of the lockdown between Wuhan and Lombardy through a psycholinguistic analysis of posts on social networks in the two weeks before and in the two weeks after the lockdown. Some of the categories that decreased in our sample between the two surveys (*leisure, home*) increased in the study of Su and colleagues (Su et al., 2020) between the pre- and post-interdiction period: in their study, the increase in the use of the category *home* between the pre- and post-interdiction period was explained by the restrictions (people did not go out, so they talked about their home more often). Instead, our results relate to two different weeks within the lockdown. We hypothesize that the decrease in words referring to *home* (e.g., *home, house, room, bed*) and *leisure* (e.g., *game, fun, play, party*) reflects a kind of habituation of the subjects to the situation of domestic isolation and that they have come to terms with the isolation and the drastic decrease in leisure activities limited exclusively to domestic activities.

Regarding the comparison of language use between men and women, it is highlighted that men used more *articles* in both time points, while women used more words related to *anxiety* (e.g., worry, fear, fearful, nervous), *social processes, past*, and *present* in T1. At T2, more words related to *positive emotions* (e.g., safe, amazing, exciting) appeared in women's texts than in men's.

These results seem to be in line with what Tausczik and Pennebaker (2010) have shown. According to these authors, women use more descriptive and emotional language with more references to others, while men use more articles. In the study by van der Vegt et al. (2020), it is clear that there are gender differences in language use with regard to the concern caused by the pandemic: in their study, women used more words expressing concern for others and fear, while men showed more concern about the social impact of the pandemic (van der Vegt et al., 2020). This aspect seems to be in line with the results of a Spanish study (Fenollar-Cortés et al., 2021) that longitudinally investigated the gender Differences in psychological impact of the confinement during the pandemia. The authors show that although the female group initially reported higher levels of negative emotions than the male group, these differences were successively reduced due to the overall improvement of the female group. Also in the study of Rodriguez-Besteiro et al. (2021) females presented a higher levels of anxiety and perception of danger than males, that showed a higher level of extraversion. However, our results aren’t confirmed by a recent Italian study (Rania and Coppola, 2021) that didn’t find any gender differences in the perception of happiness and mental health, while reported an increase of the perception of loneliness by males compared to the pre-pandemic condition.

In terms of assessing the impact of lockdown based on housing status, the literature has shown that forced coexistence had psychological impacts based on pre-pandemic levels of conflict and coping within relationship dynamics (Gambin et al., 2021); the work of Schokkenbroek et al. (2021) confirmed that isolation strained couple relationships. These assumptions may explain why participants in our sample who cohabitated with their partner used the *negative emotions* category more often than others. The category *affect*, as well as *positive sensations*, *anger*, *optimism* and *certainty*, were used more frequently by those who lived with someone (partner, family, friends/roommates) than by those who experienced the lockdown alone. In general, it has been shown that those who experienced the lockdown alone reported higher levels of distress (Raj & Bajaj 2021). Our findings seem to confirm this tendency toward hope (*optimism*) and new concrete answers (*certainty*) in the narratives of those who shared their homes and time during the lockdown.

Finally, regarding the differences between the regions of Italy, the results show that, in general, in both T1 and T2, participants from the South, especially compared with those from the Northwest, tended to describe their experiences in terms of processes and social relations (*social processes*), adopting a collective rather than individual perspective (*1st person plural*). These results seem to be consistent with the grounded theory study conducted in southern Italy (Procentese et al., 2021), which focused on the sensemaking processes resulting from the daily narratives of university students during the lockdown. Indeed, the authors showed the tendency of participants to search for meanings and for a connection between the self and others through meaningful relationships.

## Limits and conclusions

The study has some limitations, most importantly the nature of the sample: it is not homogeneous in terms of the gender of the participants. While it is representative of psychology faculties in Italy (about 70% of students are female— Conferenza dei Rettori delle Università Italiane, CRUI Group for the Gender Balance, 2021), it does not reflect all Italian university faculties; therefore, it is not possible to generalise the results. Additionally, these students may be used to deeper reflection and be more introspective than other student groups. Furthermore, socio-demographic variables that could have influenced the results, such as the economic condition, previous health conditions, any past traumatic experiences, were not taken into consideration.

Second, our surveys were conducted over a relatively short period of time: It is possible that performing the tasks after a few weeks reduced the variability of the results. In addition, because we decided to combine the texts produced over the seven days into a single text file, we lost the diary component of photo diaries, which precludes the possibility of assessing variations in the use of speech categories from one day to the next. This choice may have contributed to losing track of some psychological processes involved in the elaboration of the lockdown experience. Furthermore, the texts aggregated in this way do not allow to trace the connection with the individual photographs chosen by the students for a possible future comparative analysis.

A further limit consists in not having used any other daily psychological measures, such as anxiety or depression measures. The information collected from the use of such tools in relation to language analysis could have enriched the knowledge about the links to daily well-being.

Despite the limitations of this study, the analysis has made it possible to highlight the way in which the traumatic event due to the COVID-19 pandemic and the lockdown are put into words. It has highlighted the cognitive and emotional linguistic markers and the processes underlying the daily experiences of Italian university students. In particular, we observed that the narrative of the personal relationship between oneself, the pandemic and the state of isolation was shaped by the differences between the different parts of Italy and the different housing conditions. We believe that the opportunity to reflect and narrate daily on the pandemic experience could be useful in making sense of such an overwhelming event and allow psychologists to develop targeted support interventions. Possible future development of the study includes planning follow-up studies with the same sample to assess after some time whether and how the language used by students has changed from that used during inclusion.

## Data Availability

The data that support the findings of this study (textual corpus) are available from the corresponding author on reasonable scientific request.

## Declarations

The study was conducted in accordance with the Declaration of Helsinki, and approved by the Ethics Committee of University of Padua.
